# The expanding regulatory universe of p53 in gastrointestinal cancer

**DOI:** 10.12688/f1000research.8363.1

**Published:** 2016-04-26

**Authors:** Andrew Fesler, Ning Zhang, Jingfang Ju

**Affiliations:** 1 Translational Research Laboratory, Department of Pathology, Stony Brook University, Stony Brook, USA; 2 Department of Pharmacy, Dalian Medical University, Dalian, China

**Keywords:** p53, non-coding RNA, gastrointestinal cancer

## Abstract

Tumor suppresser gene *TP53* is one of the most frequently deleted or mutated genes in gastrointestinal cancers. As a transcription factor, p53 regulates a number of important protein coding genes to control cell cycle, cell death, DNA damage/repair, stemness, differentiation and other key cellular functions. In addition, p53 is also able to activate the expression of a number of small non-coding microRNAs (miRNAs) through direct binding to the promoter region of these miRNAs.  Many miRNAs have been identified to be potential tumor suppressors by regulating key effecter target mRNAs. Our understanding of the regulatory network of p53 has recently expanded to include long non-coding RNAs (lncRNAs). Like miRNA, lncRNAs have been found to play important roles in cancer biology.  With our increased understanding of the important functions of these non-coding RNAs and their relationship with p53, we are gaining exciting new insights into the biology and function of cells in response to various growth environment changes. In this review we summarize the current understanding of the ever expanding involvement of non-coding RNAs in the p53 regulatory network and its implications for our understanding of gastrointestinal cancer.

## Introduction

The discovery of p53 is one of the most exciting events in biological research over the past 30 years ^1– 3^. The field of p53 research represents a large growing body of exciting studies with over 75000 citations in PubMed. p53 is one of the most frequently mutated or deleted tumor suppressor genes in gastrointestinal (GI) cancers which represent nearly 30% of tumor incidences. It is well established that the classical function of tumor suppressor gene p53 is to act as a transcription factor to regulate its downstream protein coding genes in response to various growth conditions and cellular stresses ^4, 5^. Most of the research effort in the past has been devoted to the regulatory mechanism of transcriptional regulation of protein coding genes by p53. Independent of its transcriptional function, p53 is also able to regulate cell death by migrating directly to the mitochondria and interacting with B-cell lymphoma 2 (BCL-2) family member proteins to induce mitochondrial outer membrane permeability ^6^. Limited attention has been devoted to other p53 functions such as RNA binding and post-transcriptional control ^7^. With the discovery of non-coding RNA such as microRNA (miRNA), we and others have recognized the importance of post-transcriptional control mediated by non-coding RNAs in cancer ^8, 9^. Post-transcriptional and translational controls mediated by RNA binding proteins and non-coding RNAs provide cells with a great advantage in response to acute growth environment changes such as genotoxic stress caused by chemo- and/or radiation- therapy ^10– 12^. Non-coding RNAs comprise nearly 97% of transcribed RNA molecules ^13^. Much of the research efforts in the past decade concerning non-coding RNAs have been focused on short non-coding RNAs such as miRNAs and piRNAs. However, with advances in sequencing technology, now there is a growing body of evidence showing that lncRNAs also contribute to gene regulation at multiple levels ^14, 15^. Perhaps not surprisingly, important interactions have been discovered between the functions and regulation of these non-coding RNAs and p53. The relationship between non-coding RNA and p53 has been revealed to be quite dynamic, with p53 regulating the expression of some non-coding RNAs while other non-coding RNAs can function to regulate p53. While our appreciation of the important functions of non-coding RNA has grown, we have achieved a much better understanding of non-coding RNAs in the p53 regulated mechanisms in cancer.

## p53 and miRNAs

p53 is one of the most well studied tumor suppressor genes. Disruptions of p53 functions, via deletions or mutations are found in many different types of cancers, including over 50% of colorectal cancers ^16– 18^. The importance of p53 in cancers, is associated with its role as a transcriptional activator or suppressor, by which it regulates the expression of many essential genes. p53 function is crucial to maintain genome integrity and stability. p53 has been called the ‘guardian of the genome’ ^19^. It can also act as an RNA-binding protein to modulate gene expression at the post-transcriptional level. p53 binds to the 5'-UTR region of cyclin-dependent kinase 4 (CDK4) to suppress translation and it has been shown to auto-regulate its own translation by directly interacting with its own mRNA ^20, 21^.

miRNA are short non-coding RNA that are transcribed as primary miRNAs (pri-miRNA) ^22^. The pri-miRNA is cleaved by Drosha to a 70 nucleotide stem-loop pre-miRNA. Pre-miRNA is transported to the cytoplasm by Exportin 5 and further cleaved by RNAse Dicer to a 20 to 25 base pair double stranded miRNA. miRNAs modulate expression of target mRNAs by either perfect or imperfect base pairing mainly at the 3'-UTR regions of mRNA transcripts to inhibit translation and/or promote mRNA degradation. One particular miRNA can regulate multiple mRNA transcripts providing the possibility for the regulation of multiple different cellular networks and pathways by an individual miRNA ^23^. There are also multiple miRNAs that can directly interact with one particular mRNA. With the discovery of miRNAs and the fact that they can have important roles in cancer biology, as well as the well-established function of p53 in cancer, we reasoned that there may be some interplay between the two and some of these miRNAs may be involved in the p53 regulatory network. We first reported a systematic analysis of miRNA profiles in colon cancer cell lines, HCT 116, containing either wild type p53 or null p53 ^8^. In this study, we also profiled actively translated mRNAs impacted by p53 loss, and bioinformatically identified putative p53-binding sites in nearly 40% of miRNA promoter regions ( *e.g.* miR-34s, miR-192, miR-215, miR-194, miR-502, miR-200c, miR-26a, miR-15) ^8^. Many of these miRNAs were found to be directly regulated by p53 by us and other groups, thus establishing the interplay between p53 and miRNA networks in cancer ^24– 30^.

## miRNA regulation by p53

Research by us and other groups has clearly demonstrated that regulation of miRNA is among the many important functions of p53 in the cell. The miRNAs that have been shown to be regulated by p53 have important roles in regulating cellular pathways and functions such as cell cycle, apoptosis and chemoresistance. Working with the miRNAs we identified as having putative p53 binding sites in their promoter region we validated that miR-26a was directly regulated by p53 in colon cancer ^8^. miR-26a has been found to act as a tumor suppressor in mouse intestine ^31^. In gastric cancer, miR-26a also seems to act as a tumor suppressor, by targeting fibroblast growth factor 9 (FGF9) and inhibiting cell proliferation and metastasis ^32^. miR-34s are the most extensively investigated miRNAs shown to be directly regulated by p53 in a number of different tumor types ^28^. miR-34a regulation by p53 is important in p53 mediated apoptosis, with inhibition of miR-34a reducing p53-induced apoptosis ^9^. miR-34a suppresses the E2F transcription factor pathway, reducing cell cycle progression. miR-34a contributes to apoptosis regulation in colon cancer through targeting silent information regulator 1 (SIRT1). miR-34a also contributes to the activation of both p53 and p21. These functions contribute to the tumor suppressor role of miR-34a ^9, 28, 33– 35^. miR-34 is directly regulated by p53 and is reduced in 36% of human colorectal cancer tumor specimens ^33^. p53 dependent expression of miR-34 also inhibits tumor progression by disrupting an IL-6R/Stat3/miR-34a feedback loop ^36^. miR-34s have also been demonstrated to be important in other GI tumor types ^37^. In gastric cancer, miR-34 expression can activate tumor suppressor pathways in cells that lack functional p53 as well as being able to inhibit tumorsphere formation ^38^. miR-34 is one of the best characterized miRNAs that is regulated by p53 and has important functions in cancer, and thus not surprisingly, miR-34 based anti-cancer therapy also represents one of the first miRNAs to enter into clinical trials ^39^.

Beyond miR-34 as the poster child of p53 regulated miRNA, there are other important p53 regulated miRNAs. miR-192 and miR-215 have been shown by multiple groups to be regulated by p53 and their expression levels were reduced in colorectal cancer ^25, 29, 40, 41^. miR-192 and miR-215 can induce cell cycle arrest and enhance p53 mediated p21 expression when overexpressed in colon cancer cell lines. Our group has focused our efforts on investigating the roles of miR-192 and miR-215 in colorectal cancer with the interest of understanding chemoresistance mechanisms to 5-fluorouracil (5-FU) and methotrexate (MTX). We discovered that p53 and miR-192 form a positive feedback loop to regulate cell cycle and proliferation ^25^. In addition, we discovered a key protein target of miR-192 is dihydrofolate reductase (DHFR). DHFR is a protein therapeutic target of MTX. miR-192 also suppresses the expression of 5-FU protein target thymidylate synthase (TYMS, TS). These results have also been reported by another research group ^42^. However, the function of miR-215 and miR-192 seems to be different in gastric cancer. It has been reported that the expression of miR-215 is up-regulated in gastric cancer and one of the key targets is tumor suppressor retinoblastoma gene Rb1 ^43^. Consistent with this, another report shows that miR-192 and miR-215 are associated with gastric tumor invasion and lymph node metastasis ^44^. It appears that depending on the cellular and disease context, miRNAs can target different sets of mRNAs, as a result, they can function as either tumor suppressors or oncogenes. The regulatory mechanism and function of miR-192/215 will be quite unique in colorectal cancer vs. gastric cancer. One recent study demonstrated the potential of miR-192, miR-215 and miR-194 as promising detection biomarkers for Barrett's esophagus ^45^, further supporting the importance of the p53 mediated miRNAs. miR-194 has also been identified as a p53 regulated miRNA. In colon cancer, miR-194 targets thrombospondin 1 (TSP-1) and is involved in promoting angiogenesis ^46^. In gastric cancer, miR-194 has been shown to target E3 ubiquitin-protein ligase RBX1 and decrease proliferation and migration ^47^. In contrast to these miRNAs, we have identified a negative correlation between miR-502 expression and p53, suggesting that rather than inducing the expression of miR-502, p53 inhibits its expression in colon cancer. miR-502 plays a role in regulating autophagy and proliferation in colon cancer cells ^24^. miR-145 is also transcriptionally regulated by p53. miR-145 in turn suppresses the expression of cMyc and cyclin-dependent kinase 6 (CDK6), to inhibit cell proliferation and induce apoptosis ^48, 49^. miR-1204 is transcriptionally activated by p53 and also inhibits cellular proliferation ^50^.

In addition to wild type p53, mutant p53 also plays key roles in GI cancer. Studies have demonstrated that the gain-of-function of mutant p53 is an important mechanism for tumors to develop resistance and impacts tumor progression ^51, 52^. In fact, mutant p53 can directly influence miRNA expression by interacting with miRNA promoters ^53, 54^. Mutant p53 exerts oncogenic functions and promotes epithelial-mesenchymal transition (EMT) in endometrial cancer (EC) by directly binding to the promoter of miR-130b, a negative regulator of zinc finger E-box-binding homeobox 1 (ZEB-1), and inhibiting its transcription. miR-223 was recently found to be down-regulated directly by mutant p53 proteins in breast and colon cancer cell lines ^55^. Mutant p53 binds the miR-223 promoter and reduces its transcriptional activity. Such regulation requires the transcriptional repressor ZEB-1. In addition, miR-223 exogenous expression sensitizes breast and colon cancer cell lines expressing mutant p53 to treatment with DNA-damaging drugs ^55^. Let-7i has also been found to be regulated by mutant p53, inhibiting invasion and migration ^54^. These results suggest that it will be important to identify additional miRNAs that are regulated by various mutant p53 proteins. Table 1 summarizes some p53 regulated miRNAs in GI cancer.

**Table 1.** p53 regulated miRNA in GI cancer.

miRNA	Wtp53/Mutp53	Transcriptional Target	Tumor Suppressor/ Oncogene	Function	Ref.
miR-26a	Wt	Yes	Tumor Suppressor	↓Proliferation ↓Metastasis ↑Apoptosis	8, 31, 32
miR-34	Wt	Yes	Tumor Suppressor	↓Proliferation, ↑Cell Cycle Arrest, ↑Apoptosis, ↑Senescence, ↓Tumor Sphere Formation, ↓EMT	9, 28, 33– 35, 38, 110
miR-192/ miR-215	Wt	Yes	*	↑/↓Proliferation, ↑/↓Chemosensitivity, ↑Cell Cycle Arrest	25, 29, 42, 44
miR-194	Wt	Yes	*	↓Proliferation, ↓Migration/ Invasion, ↑Angiogenesis	46, 47
miR-502	Wt	No	Tumor Suppressor	↓Autophagy, ↑Cell Cycle Arrest	24
miR-145	Wt	Yes	Tumor Suppressor	↓Proliferation	48, 49
miR-1204	Wt	Yes	Tumor Suppressor	↑Cell Cycle Arrest, ↑Apoptosis	50
miR-130b	Mut	No	Tumor Suppressor	↓EMT	53
miR-223	Mut	No	Tumor Suppressor	↓Chemoresistance	55
Let-7i	Mut	No	Tumor Suppressor	↓Migration/Invasion, ↓Metastasis	54

*--Role not clear, or conflicting reports in different cancer types

## p53 regulation by miRNA

The relationship between p53 and miRNAs is more complex than just transcription regulation by p53. In fact, the interaction between the two is a two way street, with several miRNAs being able to regulate p53 expression either through direct targeting, or through regulation of other proteins that in turn modulate p53 expression and function. Some of the miRNAs regulated by p53 are actually able to act in feedback loops to regulate p53 as well. We investigated the regulation mechanism of p53 by miR-215 in colorectal cancer and discovered that a key target of miR-215 is denticleless protein homolog (DTL). The suppression of DTL by miR-215 triggered an up-regulation of p53 and p21 ^26^. DTL (RAMP, CDT2) is thought to play an essential role in DNA synthesis, cell cycle progression, proliferation and differentiation ^56^. DTL controls cell cycle progression through several different mechanisms, and has an important role in the early radiation induced G2/M checkpoint ^57, 58^. The Proliferating cell nuclear antigen (PCNA)-coupled CUL4/DDB1/DTL complex can ubiquitinate and degrade key cell cycle proteins such as p53, mouse double minute 2 homolog (MDM2), p21, and E2F1 ^59– 61^. miR-502, also regulated by p53, acts in a feedback loop to repress expression of p53 indirectly ^24^. miR-34 also acts in a feedback loop with p53 in colon cancer cells. Transfection of miR-34 into colon cancer cells leads to an increase in p53 and p21 expression as a result of down regulation of the E2F pathway ^33^. Several other miRNAs including miR-339-5p and miR-542-3p positively regulate p53 through their targeting of p53 inhibitor MDM2 ^62, 63^. In addition to these miRNAs that regulate p53 through indirect mechanisms, others have been found to directly target p53. Among the first miRNAs found to target p53 directly, were miR-125b and miR-504 ^64, 65^. miR-504 was demonstrated to target p53 in several cancer types, and reduce *in vivo* tumor growth of colon cancer cells ^65^. In metastatic gastric cancer, miR-300 is up-regulated and acts as a tumor promoter. miR-300 was found to directly target p53 by interacting with the 3'-UTR of p53. Overexpression of miR-300 led to decreased p53 expression in gastric cancer cells, and inhibition of miR-300 led to an increase in p53 expression. Overexpression of p53 also reduced tumor promotion by miR-300, highlighting the importance of p53 targeting in miR-300 cellular function ^66^. Additionally, miR-25 and miR-30d directly targeted p53 to regulate apoptosis in colon cancer cells ^67^. These results suggest that not only can the functions of miRNAs be modulated by the p53 status in colorectal cancer, the tumor suppressive function of p53 can also be modulated by the post-transcriptional controls of various miRNAs under different stress and/or physiological conditions, providing p53 with a greater flexibility to control cell cycle and cell death.

**Figure 1.**
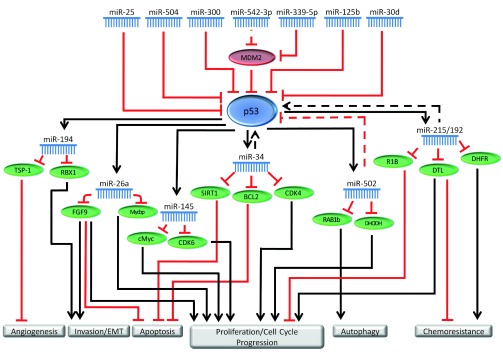
miRNAs have important functions in the p53 regulatory network. miRNAs regulate p53 through direct targeting as well through indirect mechanisms such as targeting p53 regulators. p53 also regulates several miRNAs, through transcriptional activation as well as other mechanisms. The miRNAs involved in the p53 network carry out important functions regulating several cellular pathways such as proliferation, apoptosis, invasion and migration. In this figure, solid lines represent direct regulation, while dashed lines represent indirect, or poorly characterized regulation.

Clearly miRNAs play important roles in the p53 regulatory network in GI cancer. p53 can regulate the transcription of several miRNAs that have important cellular functions in GI cancer. In addition, several miRNAs can regulate p53 expression to influence cellular pathways in cancer. The importance of the role of miRNAs in the p53 network is reflected in several other reviews that highlight this interaction ^68– 72^. Our understanding of these networks will likely continue to increase as we expand our understanding of the important functions of miRNAs as well as the roles of other non-coding RNAs in p53 regulation and function. Table 2 summarizes some miRNAs that regulate p53 in GI cancers. Figure 1 depicts the involvement of miRNAs in the p53 regulatory network. 

**Table 2.** miRNAs that regulate p53 in GI Cancer.

miRNA	Regulation +/-	Direct/ Indirect	Function	Ref.
miR-215	+	Indirect	↑/↓Proliferation, ↑/↓Chemosensitivity, ↑Cell Cycle Arrest	25, 29, 42, 44
miR-502	-	Indirect	↓Autophagy, ↑Cell Cycle Arrest	24
miR-34	+	Indirect	↓Proliferation, ↑Cell Cycle Arrest, ↑Apoptosis, ↑Senescence, ↓Tumor Sphere Formation, ↓EMT	9, 28, 110
miR-339-5p	+	Indirect	↑Cell Cycle Arrest, ↑Senescence, ↓Proliferation	62
miR-542-3p	+	Indirect	↑Cell Cycle Arrest	63
miR-125b	-	Direct	↓Apoptosis	64
miR-504	-	Direct	↓Apoptosis, ↓Cell Cycle Arrest	65
miR-300	-	Direct	↑Proliferation, ↑Migration	66
miR-25	-	Direct	↓Apoptosis, ↓Cell Cycle Arrest, ↓Senescence	67
miR-30d	-	Direct	↓Apoptosis, ↓Cell Cycle Arrest, ↓Senescence	67

## p53 and lncRNAs

Interaction between p53 and non-coding RNA is certainly not limited to miRNA. Recent evidence has demonstrated that lncRNAs also have important functions in the p53 regulatory network. LncRNAs (200 nucleotides or more in length), thanks to improvements in sequencing technology, have begun to emerge recently as critical regulatory RNAs ^73– 75^. The understanding of the roles of lncRNAs in diseases, such as cancer, is still very limited but recent work has shown that these molecules can have some important functions in cancer biology and like miRNA are tied into the p53 regulatory network.

## LncRNAs regulated by p53

The field of lncRNA research remains in its early stages, and we are still identifying more lncRNAs and discovering the important functions they have in the cell. The progress that has been made thus far is quite interesting and encourages increased investigation. A systematic ChIP-Seq analysis has identified 23 lncRNAs that are up-regulated by p53 ^76^. Among the over six thousand lncRNAs that have been identified, lincRNA-p21 is one of the better characterized lncRNAs and importantly, is regulated by p53 ^77, 78^. LincRNA-p21 locates next to the p21 gene on mouse chromosome 17 and is activated upon DNA damaging signals in mouse cells. It has been reported that lincRNA-p21 is a direct transcriptional target of p53 and is involved in p53-dependent transcriptional responses. There is a significant overlap among the genes regulated by lincRNA-p21 expression and genes that are repressed by p53. Heterogeneous nuclear ribonucleoprotein K (hnRNP-K) plays an important role in the ability of lincRNA-p21 to regulate these genes. Expression of lincRNA-p21 in mouse embryonic fibroblasts (MEFs) also has shown the ability to induce apoptosis ^77^. Subsequent studies further revealed that lincRNA-p21 activates p21 in cis to promote polycomb target gene expression and to enforce the G1/S checkpoint ^79^. Recent studies also demonstrated that in human cervical carcinoma HeLa cells, a RNA binding protein, human antigen R (HuR), modulates the expression level of lincRNA-p21, which in turn regulates its target protein translation, such as transcription factor jun-B (JUNB) and β-catenin ^78^. Intriguingly for this review, lincRNA-p21 can be regulated by some miRNAs including let-7 ^80^. Our group has recently show that lincRNA-p21 is associated with colorectal cancer progression ^81^. Such association may be due to the unique function of lincRNA-p21 under hypoxia. LincRNA-p21 is a hypoxia-responsive lncRNA and is essential for hypoxia-enhanced glycolysis ^82^. There is a positive feedback loop between hypoxia-inducible factor 1-alpha (HIF-1α) and lincRNA-p21 to promote tumor growth and the regulation of the Warburg effect. lincRNA-p21 has also been found to regulate the Wnt/β-catenin signaling pathway, and be associated with susceptibility to radiation therapy in colon cancer ^83^. LincRNA-p21 is a powerful example of a long non-coding RNA, regulated by p53 that carries out important functions in the response pathway of p53. Another p53 regulated lncRNA named, p53 induced noncoding transcript (Pint), is a direct transcriptional target of p53. Pint is a nuclear RNA, that directly interacts with polycomb repressive complex 2 (PRC2), and is required for PRC2 targeting of specific genes for H3K27 tri-methylation and repression ^84^. Pint is down-regulated in primary colon tumors and overexpression of Pint inhibits tumor cell proliferation, suggesting a potential tumor suppressor role ^84^. Tumor suppressor candidate 7 (Tusc7) (LncRNA loc285194) has also been shown to be a p53 mediated tumor suppressor in colon cancer ^85^. Tusc7 is transcriptionally activated by p53 to inhibit cell growth and exerts its function by suppressing miR-211 ^85^. In patient samples, Tusc7 was shown to be reduced in cancer compared to normal colon tissue. Reduced Tusc7 expression is associated with increased tumor size, stage and distant metastasis as well as decreased survival ^86^. Similar results were found in esophageal cancer as well as pancreatic cancer, suggesting Tusc7 might be a good biomarker candidate ^87, 88^. In gastric cancer, Tusc7 expression is reduced in patient samples, and in cell lines decreases tumor cell growth. Tusc7 expression is also induced by wild type p53 but not mutant p53 ^89^. While Tusc7 seems to act as a tumor suppressor and is reduced in several types of cancer, the picture for taurine up-regulated 1 (Tug1), another p53 regulated lncRNA, is not as clear. Tug1 was first discovered to be important in retinal development and was then shown to be a direct transcriptional target of p53 in the context of non-small cell lung cancer ^90, 91^. The role of Tug1 in cancer however, seems to be different in different cellular contexts. In lung cancer, Tug1 expression was found to be decreased in cancer tissue compared to normal. Lower expression of Tug1 correlates with higher tumor stage, increased tumor size and decreased overall survival ^91^. In esophageal cancer however, the role of Tug1 seems to be quite different. Tug1 is found to be over expressed in cancer tissue with expression being correlated with tumor stage. Knockdown of Tug1 also seems to inhibit cancer cell proliferation as well as migration ^92^. This oncogenic type function for Tug1 has also been found in bladder cancer where it appears to be up-regulated in cancer and promote cancer invasion as well as resistance to radiotherapy ^93^. There is clearly a need to perform more research on Tug1 to get a more in-depth understanding of its functions, and confirm what has been found in these different types of cancer. The disparity seen thus far however, may be due to differences in functions of this lncRNA in different cellular contexts, something that may be expected based on what we have already discovered about the functions of miRNA in cancer. LncRNA activator of enhancer domains (LED) has recently been identified via genome-wide profiling as a p53 induced lncRNAs that acts as an enhancer to regulate p21 ^94^. LED knockdown reduces p21 enhancer induction, activity, and cell cycle arrest following p53 activation. LED was identified and its function assessed in MCF-7 cells, however it has also been identified in a genome wide profile of colon cancer cells, though its specific function in this cellular context will need to be investigated ^95^. Also identified in genome wide screening in colon cancer cells, PR-lncRNA-1 and PR-lncRNA-10 were identified as transcriptional targets of p53, that then act to regulate the transcription of target genes. These lncRNAs, may have potential tumor suppressor like function, and seem to play a role in regulating p53 anti-apoptotic and cell cycle regulatory functions. They may be important lncRNAs to investigate further ^96^. Perhaps one of the more interesting p53 regulated lncRNAs is PVT1, which is transcriptionally induced by p53. Evidence suggests that PVT1 has an anti-apoptotic effect in colon cancer cells, and promotes proliferation and invasion ^50, 97^. PVT1 expression is also increased in colon cancer patients and increased expression predicts poor prognosis. At the same time, miR-1204 is also encoded from the PVT1 locus and seems to increase apoptosis and inhibit cell cycle progression ^50^. This demonstrates the complex and dynamic nature of the relationship between p53 and non-coding RNAs. Linc-Regulator Of Reprogramming (Linc-ROR) is a transcriptional target of p53, and inhibits p53 related apoptosis and cell cycle arrest ^98^. Beyond GI cancers, PANDA has been identified as a lncRNA that is a direct transcriptional target of p53. However, its function in GI cancers has not been investigated. This is something that needs to be further investigated as PANDA may have some roles in regulating apoptosis and cell cycle arrest in the p53 pathway ^99^. Table 3 summarizes the p53 regulated lncRNAs based on their critical molecular and cellular functions in GI cancers.

**Table 3.** p53 Regulated lncRNA in GI Cancer.

lncRNA	Transcriptional Target	Tumor Suppressor/ Oncogene	Function	Ref.
lincRNA-p21	Yes	*	↑Apoptosis, ↑Cell Cycle Arrest, ↑Radiation Sensitivity, ↑Hypoxia Resistance	77, 79, 81– 83
Pint	Yes	Tumor Suppressor	↑Apoptosis, ↓Proliferation	84
Tusc7	Yes	Tumor Suppressor	↓Proliferation,	85– 89
Tug1	Yes	*	↑Proliferation, ↑Migration/Invasion, ↑EMT, ↑Radiation Resistance	91– 93
LED	Yes	*	↑Cell Cycle Arrest	94, 95
PR-lncRNA-1	Yes	Tumor Suppressor	↑Apoptosis, ↓Proliferation	96
PR-lncRNA-10	Yes	Tumor Suppressor	↑Apoptosis, ↓Proliferation	96
PVT1	Yes	*	↑Proliferation ↓Apoptosis	50, 97
LincRNA-ROR	Yes	*	↓Apoptosis, ↓Cell Cycle Arrest	98, 105, 106

*--Role not clear, or conflicting reports in different cancer types

## p53 regulation by lncRNA

**Figure 2.**
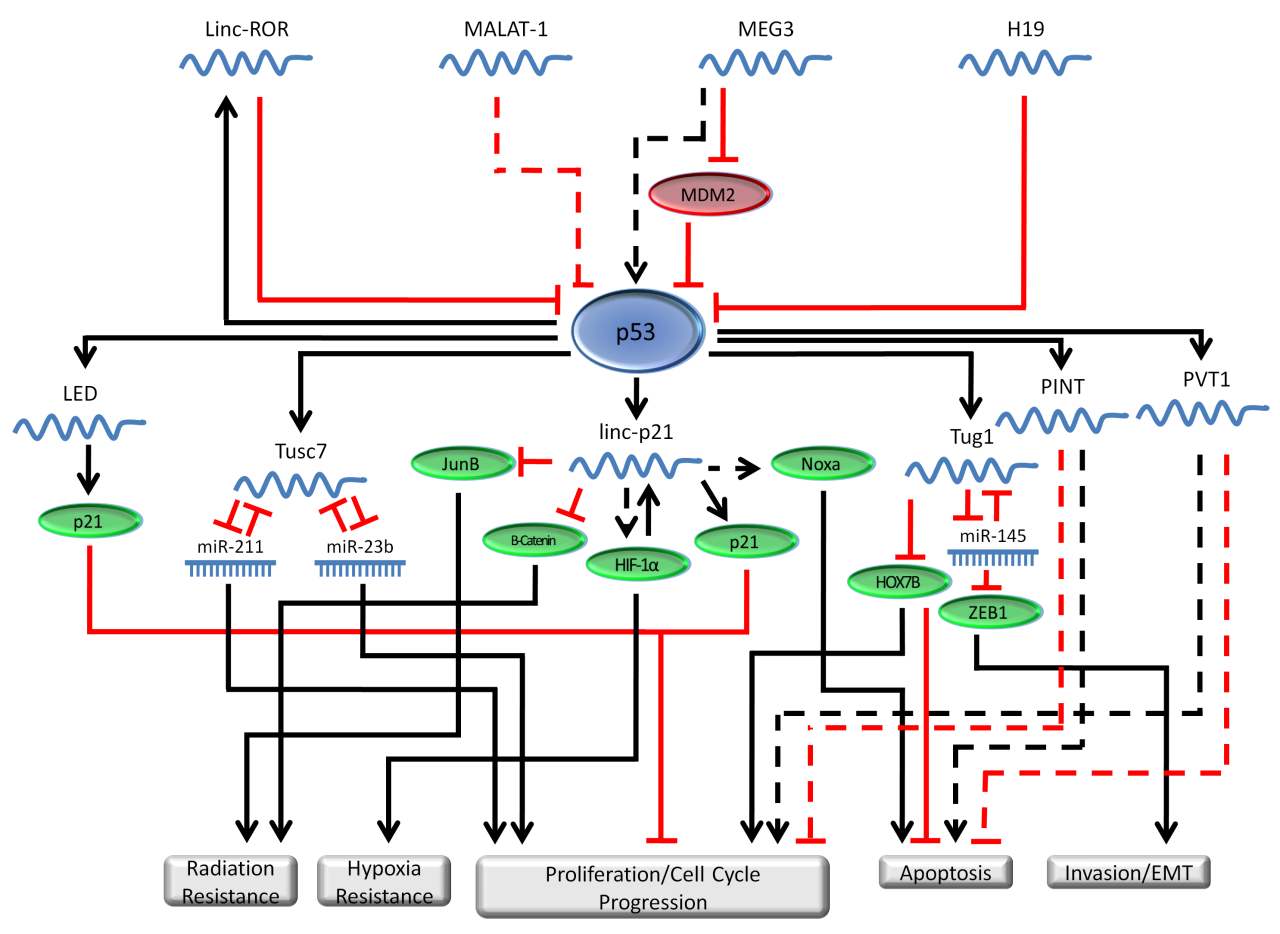
lncRNA have important functions in the p53 regulatory network. lncRNAs, have a function in regulating p53. p53 in turn regulates the expression of several different lncRNAs. The lncRNAs involved in the p53 network regulate cellular functions such as proliferation, apoptosis, invasion and migration. In this figure, solid lines represent direct regulation, while dashed lines represent indirect, or poorly characterized regulation.

Clearly there are quite a few lncRNA that are regulated by p53 that are already known to play important roles in cancer, and undoubtedly more will be discovered in the near future. Like miRNAs however, the relationship between p53 and lncRNAs works both ways, and there have been several lncRNAs discovered to regulate p53 as well. LncRNAs can function as modulators by preventing p53 degradation. One example is human maternally expressed gene 3 (MEG3). MEG3 is a non-coding RNA that functions as a tumor suppressor in colon cancer cell lines. MEG3 down-regulates MDM2, which in turn up-regulates p53 expression level ^100^. MEG3 can inhibit cell proliferation in the absence of p53, suggesting a possible p53 independent tumor suppressor role. LncRNAs can also inactivate p53 function and H19 is an example. The H19 lncRNA has been demonstrated to be associated with p53 in gastric cancer ^101^. Such interaction resulted in partial inactivation of p53. Metastasis associated lung adenocarcinoma transcript 1 (MALAT-1) is another lncRNA that seems to regulate p53. In the case of MALAT-1, it seems to be a negative regulator of p53, and depletion of MALAT-1 leads to an increase in p53 expression ^102^. In colon cancer, MALAT-1 has increased expression in cancer tissue vs. normal. Increased MALAT-1 is associated with poor patient prognosis ^103^. In colon cancer cell lines, overexpression of MALAT-1 promotes proliferation, migration and invasion. These functions are associated with regulation of A-kinase anchor protein 9 (AKAP-9) by MALAT-1 ^104^. LincRNA-ROR acting in a feedback loop, is also able to regulate p53, and knockdown of lincRNA-ROR leads to an increase in genes in the p53 pathway response ^98, 105^. There is also recent evidence that lincRNA-ROR expression is reduced in colon cancer, however more needs to be done to investigate this role ^106^. Table 4 summarizes lncRNAs that regulate p53 in GI cancers. Through their regulation by p53 or their ability to regulate p53, lncRNAs clearly have important functions in the p53 network, and our appreciation of these roles will continue to grow as we discover additional lncRNAs and elucidate their functions in cancer. Figure 2 depicts lncRNAs’ roles in the p53 regulatory network.

**Table 4.** lncRNA that regulate p53 in GI Cancer.

lncRNA	Regulation +/-	Tumor Suppressor/ Oncogene	Function	Ref.
MEG3	+	Tumor Suppressor	↓Proliferation	100
H19	-	Oncogene	↑Proliferation, ↓Apoptosis	101
MALAT-1	-	Oncogene	↑Cell Cycle Progression, ↑Migration/Invasion	102– 104
LincRNA-ROR	-	*	↓Apoptosis, ↓Cell Cycle Arrest	98, 105, 106

*--Role not clear, or conflicting reports in different cancer types

## Summary

The research community continues to push the boundaries of the p53 regulatory networks beyond protein coding genes to non-coding RNAs and other novel entities. There are many circular RNAs that have been discovered recently ^107, 108^. The impact of p53 on circular RNAs in GI cancer will potentially be an important field to explore going forward. As protein coding genes only represent a small percentage of our genome, we can expect more exciting discoveries in the non-coding RNA field impacted by p53. We hope that with the advancement of high throughput genomics technology and computational biology approaches, we can fully access the complete spectrum and scope of the p53 regulatory network. Such insight will provide a foundation to study other key proteins in cancer and other diseases. It will also help us to develop novel therapeutic strategies to combat cancer ^109^.
