# Supplementary material for: The expanding regulatory universe of p53 in gastrointestinal cancer
Source: F1000Res. 2016 Apr 26;5:756. [Version 1] doi: 10.12688/f1000research.8363.1 (PMC4963207; doi:10.12688/f1000research.8363.1)
Supplement: Peer review history

# Peer review: Referee response for version 1

**DOI:** 10.5256/f1000research.8996.r14211

**Published:** 2016-06-07

**Authors:** Rajeev Samant

**Affiliations:** 1 Department of Pathology, University of Alabama at Birmingham, Birmingham, AL, USA

This article by Fesler *et al. *is a timely summary of the state of the art of P53 related regulatory mechanisms with specific emphasis on colon cancer. P53 is certainly the most critical node of oncogenesis and progression. With the rapidly advancing information about the importance of non-coding RNA in the field of cancer biology it is critical to establish an integrated picture that allows us to make working models for regulation. The review has done a perfect job towards this by providing two well designed figures. Overall it is a very timely review of this ever evolving field. It is really appreciated that the authors have provided a bit of introduction on what are miRNAs and what are Lnc RNAs. It will make it easy for readers that are new to this field to appreciate the review.

 It is intriguing that the authors have provided a glimpse of future directions for the field. Overall I do not have any major or necessary suggestions. However I do have a curious request. Authors may wish to provide insight on if there is any way to integrate the miRNA based pathways to mRNA based pathways and if that has been done for P53 field.    

I have read this submission. I believe that I have an appropriate level of expertise to confirm that it is of an acceptable scientific standard.

# Peer review: Referee response for version 1

**DOI:** 10.5256/f1000research.8996.r13581

**Published:** 2016-05-20

**Authors:** Yuichiro Tanaka

**Affiliations:** 1 Department of Urology, San Francisco Veterans Affairs Medical Center, University of California, San Francisco, San Francisco, CA, USA

In this review, the authors systematically summarize the interplay between non-coding RNAs and the P53 pathway, with emphasis on how this affects gastrointestinal cancer. The non-coding RNAs are focused on miRNA and lncRNA, and how these both regulate and get regulated by P53.

 The review is solid. The authors highlight each of the major miRNA or lncRNA involved with p53 in gastrointestinal cancer in a concise way. Mutant p53 and its effects on these noncoding RNA’s are also discussed. They go into regulation of noncoding RNA or P53, not only by direct but indirect mechanisms and include genes involved in this regulation. They also touch up on noncoding RNA that have opposite effects depending on cancer type and thus, not biased. References are appropriate. The use of tables and figures highlight the key points made for each of the miRNA’s and lncRNA’s and thus, make it easier for the reader to follow.

 Comment: The summary section is not really a summary of what was discussed. It is more of forthcoming ideas. Recommend adding a few sentences or a paragraph summarizing what was actually discussed since this is a summary section. This can then be followed by what is currently stated such as circular RNA, genomics technology and computational approaches, and the rest, as future approaches. Alternatively, the heading could be changed to “Future approaches” or something similar, if permissible.

I have read this submission. I believe that I have an appropriate level of expertise to confirm that it is of an acceptable scientific standard.
